# Emerging Biomarkers in Renal Damage

**DOI:** 10.1155/2014/893068

**Published:** 2014-09-03

**Authors:** Pasquale Ditonno, Cees van Kooten, Loreto Gesualdo, Giuseppe Grandaliano, Giuseppe Lucarelli

**Affiliations:** ^1^Department of Emergency and Organ Transplantation, Urology, Andrology and Kidney Transplantation Unit, University of Bari, Piazza G. Cesare 11, 70124 Bari, Italy; ^2^Department of Nephrology, Leiden University Medical Center, Albinusdreef 2, P.O. Box 9600, 2300 RC Leiden, The Netherlands; ^3^Nephrology, Dialysis and Transplantation Unit, Department of Medical and Surgical Sciences, University of Foggia, Viale L. Pinto 1, 71122 Foggia, Italy; ^4^Nephrology, Dialysis and Transplantation Unit, Department of Emergency and Organ Transplantation, University of Bari, Piazza G. Cesare 11, 70124 Bari, Italy

Acute kidney injury (AKI) is a devastating clinical condition strongly associated with increased morbidity and mortality in critically ill patients. Traditional methods of identifying kidney injury, through measurement of blood urea nitrogen and serum creatinine, are problematic in that they are slow to detect decreases in glomerular filtration rate (GFR) and are influenced by a variety of factors that are not related to GFR changes. Many genes are upregulated in the damaged kidney with the corresponding protein products appearing in plasma and urine. Some of these are candidate markers for more timely diagnosis of AKI. An ideal biomarker of kidney injury would be a substance that the kidney releases immediately in response to damage and that can be detected in the blood or urine without significant metabolism. In recent years, the the introduction of high-throughput omics technologies has led to identification of new biomarkers of renal damage with more favorable test characteristics than creatinine. Advances in this field of research are based on a more detailed understanding of the fundamental biological mechanisms involved in the renal damage progression, as well as on advances in genomic, transcriptomic, proteomic, and metabolomic research.

The purpose of this special issue is to present original research and review articles that provide new insights into molecular pathology underlying the acute and chronic kidney injury and identify novel diagnostic and prognostic biomarkers for these clinical conditions.

Many studies have explored the molecular events associated with the development of tubular atrophy and interstitial fibrosis induced by chronic urinary tract obstruction. Moreover it is well known that the recovery of renal function after relief of ureteral obstruction depends on several factors including the location and duration of the obstruction, whether it is complete or partial, time before relief, and the presence of infection [1]. In their article, G. Lucarelli et al. summarize the role of the emerging urinary biomarkers of obstructive nephropathy based on the current understanding of the pathophysiology of renal injury.

Drug-induced nephrotoxicity plays an important role in the high prevalence and incidence of AKI, especially in neonatal intensive care units. In preterm newborns, one of the most important factors causing the pathogenesis and the progression of AKI is the interaction between the individual genetic code, the environment, the gestational age, and the disease. In this contest, M. Mussap et al. provide a broad overview of the current applications of metabolomics and novel biomarkers for assessing drug-induced toxic nephropathy and AKI in neonatology.

Complement activation is an important mechanism of renal injury in different diseases and in particular in kidney damage associated with ischemia-reperfusion [2–5]. In this scenario, E. Rodríguez et al. showed that complement pathway was activated in AKI, regardless of the etiology of AKI, leading to the production of lytic complex C5b-C9. Moreover plasmatic membrane attack complex concentrations identified AKI patients at risk of developing serious outcomes like death during hospitalization or unrecovered renal function at time of hospital discharge.

Nowadays the association of a calcineurin inhibitor (CNI) with mycophenolate mofetil (MMF) represents the backbone of solid-organ transplant immunosuppression. Although CNIs (cyclosporine A (CsA) and tacrolimus (FK506)) remain the most effective and widely used immunosuppressive agents in organ transplantation, their prolonged use may result in renal toxicity, renal dysfunction, and irreversible renal failure characterized by extensive tubulointerstitial fibrosis. To minimize the CNIs-associated nephrotoxicity, alternative protocols have been introduced especially with the increasing use of suboptimal donor organs [6–8]. One of these strategies includes the conversion from CsA to other drugs, especially sirolimus (SRL), an inhibitor of the mammalian target of rapamycin (mTOR). Using an experimental model to clarify the pathways of nephropathy evolution in a protocol of conversion from CsA to SRL, J. Sereno et al. demonstrated how conversion to SRL prevented CsA-induced renal damage evolution. Moreover these authors showed that prolonged CsA exposure aggravated renal damage, without clear changes on the traditional markers, but with changes in serums TGF-*β* and IL-7 and kidney TGF-*β* and mTOR.

These and other studies published in this special issue underline the need for new diagnostic and prognostic biomarkers. The discovery and validation of biomarkers for AKI will improve the early diagnosis of tubular injury, thereby facilitating timely therapeutic intervention. Novel, sensitive biomarkers should also help to differentiate between etiologies of AKI, predict the severity of renal damage, and provide a tool for differentiating between patients who would benefit from early initiation of renal replacement therapy and those from whom support should be withheld or withdrawn.

*Pasquale Ditonno* *Cees van Kooten* *Loreto Gesualdo* *Giuseppe Grandaliano* *Giuseppe Lucarelli*
